# Bovine tumor necrosis factor-alpha Increases IL-6, IL-8, and PGE2 in bovine fibroblast-like synoviocytes by metabolic reprogramming

**DOI:** 10.1038/s41598-023-29851-y

**Published:** 2023-02-24

**Authors:** Carolina Manosalva, Pablo Alarcon, John Quiroga, Stefanie Teuber, Maria D. Carretta, Hedie Bustamante, Rodrigo Lopez-Muñoz, Maria A. Hidalgo, Rafael A. Burgos

**Affiliations:** 1 Institute of Pharmacy, Faculty of Sciences, Universidad Austral de Chile, Valdivia, Chile; 2 Laboratory of Immunometabolism, Institute of Pharmacology and Morphophysiology, Faculty of Veterinary Sciences, Universidad Austral de Chile, Valdivia, Chile; 3 Veterinary Clinical Sciences Institute, Faculty of Veterinary Sciences, Universidad Austral de Chile, Valdivia, Chile

**Keywords:** Inflammation, Cell biology, Immunology, Diseases, Pathogenesis

## Abstract

Lameness is a common condition in dairy cattle caused by infectious or noninfectious agents. Joint lesions are the second most common cause of lameness and can be diagnosed in association with the presentation of digit injuries. Fibroblast-like synoviocyte (FLS) are predominant cells of synovia and play a key role in the pathophysiology of joint diseases, thus increasing the expression of proinflammatory mediators. Tumor necrosis factor-alpha (TNF-α) is a potent proinflammatory cytokine involved in cyclooxygenase 2 (COX-2) and proinflammatory cytokine expression in FLS. Previously, TNF-α was demonstrated to increase hypoxia-inducible Factor 1 (HIF-1), a transcription factor that rewires cellular metabolism and increases the expression of interleukin (IL)-6 in bovine FLS (bFLS). Despite this, the proinflammatory effects of TNF-α in bFLS on metabolic reprogramming have been poorly studied. We hypothesized that TNF-α increases glycolysis and in this way controls the expression of IL-6, IL-8, and COX-2 in bFLS. Results first, gas chromatography/mass spectrometry (GC/MS)-based untargeted metabolomics revealed that bTNF-α altered the metabolism of bFLS, increasing glucose, isoleucine, leucine, methionine, valine, tyrosine, and lysine and decreasing malate, fumarate, α-ketoglutarate, stearate, palmitate, laurate, aspartate, and alanine. In addition, metabolic flux analysis using D-glucose-^13^C_6_ demonstrated an increase of pyruvate and a reduction in malate and citrate levels, suggesting a decreased flux toward the tricarboxylic acid cycle after bTNF-α stimulation. However, bTNF-α increased lactate dehydrogenase subunit A (LDHA), IL-6, IL-8, IL-1β and COX-2 expression, which was dependent on glycolysis and the PI3K/Akt pathway. The use of FX11 and dichloroacetate (DCA), an inhibitor of LDHA and pyruvate dehydrogenase kinase (PDK) respectively, partially reduced the expression of IL-6. Our results suggest that bTNF-α induces metabolic reprogramming that favors glycolysis in bFLS and increases IL-6, IL-8, IL-1β and COX-2/PGE2.

## Introduction

Joint inflammation is the second most common cause of lameness in cattle^1^. The lesions affecting the joint are mainly hock lesions caused by mechanical injuries, fermentative digestive disturbances, or septic origin^1–3^. In cattle with acute ruminal acidosis, aseptic polysynovitis characterized by a massive presence of polymorphonuclear cells in the synovial fluid has been described^3,4^. The synovial fluid is secreted by the synovial membrane (synovium), which lines the articular capsule. Fibroblast-like synoviocyte (FLS) are the predominant cell types of synovial intima^5^ and assure the structural and physiological integrity of diarthrodial joints, regulating the synovial fluid composition and the extracellular matrix of the joint lining^6^. FLS is also key in the pathophysiology of synovitis-arthritis due to its ability to express several proinflammatory mediators^7,8^. In this way, FLS could be involved in the pathogenesis of cattle lameness associated with arthritis^9,10^. In support of this scheme, in cattle with severe symptoms of laminitis, an increase in albumin and globulins in synovial fluid has been observed, suggesting that arthritis and laminitis might be clinical manifestations of a common etiology^11^.

Tumor necrosis factor alpha (TNF-α) is one of the most prominent inflammatory markers found in synovial fluid^12^. During the transition period, serum concentrations of TNF-α tended to be greater in cows with lameness than in healthy animals^13^. An increase in serum TNF-α has been described in lame cattle in response to foot rot^14^; moreover, in vitro LPS stimulation can increase the release of TNF-α in bovine hoof dermal cells, supporting a potential role of this cytokine in laminitis^15^. TNF-α is produced by activated macrophages and T cells and exerts proinflammatory effects, inducing the expression of IL-8, IL-6 and COX-2 in human FLS^16–19^. Recently, we demonstrated that bovine TNF-α induces the expression of proinflammatory cytokines such as IL-6 and IL-8 via the NF-κB pathway in bovine FLS (bFLS)^20^.

FLS isolated from RA patients show higher glycolytic activity^17^. In fact, the increased expression of glycolytic enzymes, such as HK2, which catalyze the first step in glucose metabolism, PFKFB, which plays a critical role in regulating glycolysis in the cytoplasm, and PDK1, a key enzyme that acts between glycolysis and tricarboxylic acid (TCA) cycle, supporting the idea that increased glycolysis could induce a more aggressive FLS phenotype^17,21–23^.

We previously observed the presence of synovitis in heifers with acute ruminal acidosis and an increase in pyruvic acid and lactic acid in synovial fluid, suggesting that glycolysis could be altered in the inflamed joint^10^. D-Lactate is augmented during acute ruminal acidosis and induces metabolic reprogramming in bFLS, enhancing glycolysis and gluconeogenesis, and pyruvate, and galactose metabolism, which is attributable to an increase in the HIF-1 pathway^24^. Similarly, the role of the HIF-1 pathway in TNF-α-induced IL-6 expression in bFLS has been proposed^24^. HIF-1 is a transcription factor that controls the expression of genes related to inflammation, angiogenesis, and energy metabolism^25,26^. These results suggest that TNF-α could favor metabolic rewiring in bovine FLS increasing glycolysis and proinflammatory gene expression. In rheumatoid arthritis-FLS (RA-FLS), TNF-α induces several metabolic changes, including amino acid biosynthesis, purine metabolism, fatty acid metabolism and glycolysis^27^. Moreover, the increase in glycolysis has been demonstrated to be key for IL-6, IL-8, CCL-2 and CXCL-10 expression induced by TNF-α in RA-FLS^17^. Despite these results, information about the role of glycolysis in the effects of bovine TNF-α (bTNF-α) in bFLS is limited. In the present work, we tested the hypothesis that bTNF-α can favor metabolic rewiring, favoring glycolysis and the expression of IL-6, IL-8 and COX-2 in bFLS.

## Results

### bTNF-α-induced metabolic changes in bFLS

The metabolites obtained from control bFLS and bFLS treated with bTNF-α were detected by GC/MS untargeted metabolomic analysis. More than 1000 unique m/z values with retention times were detected. By deconvolution and alignment using MS-DIAL software, a total of 88 metabolites were identified.

**Figure 1:**
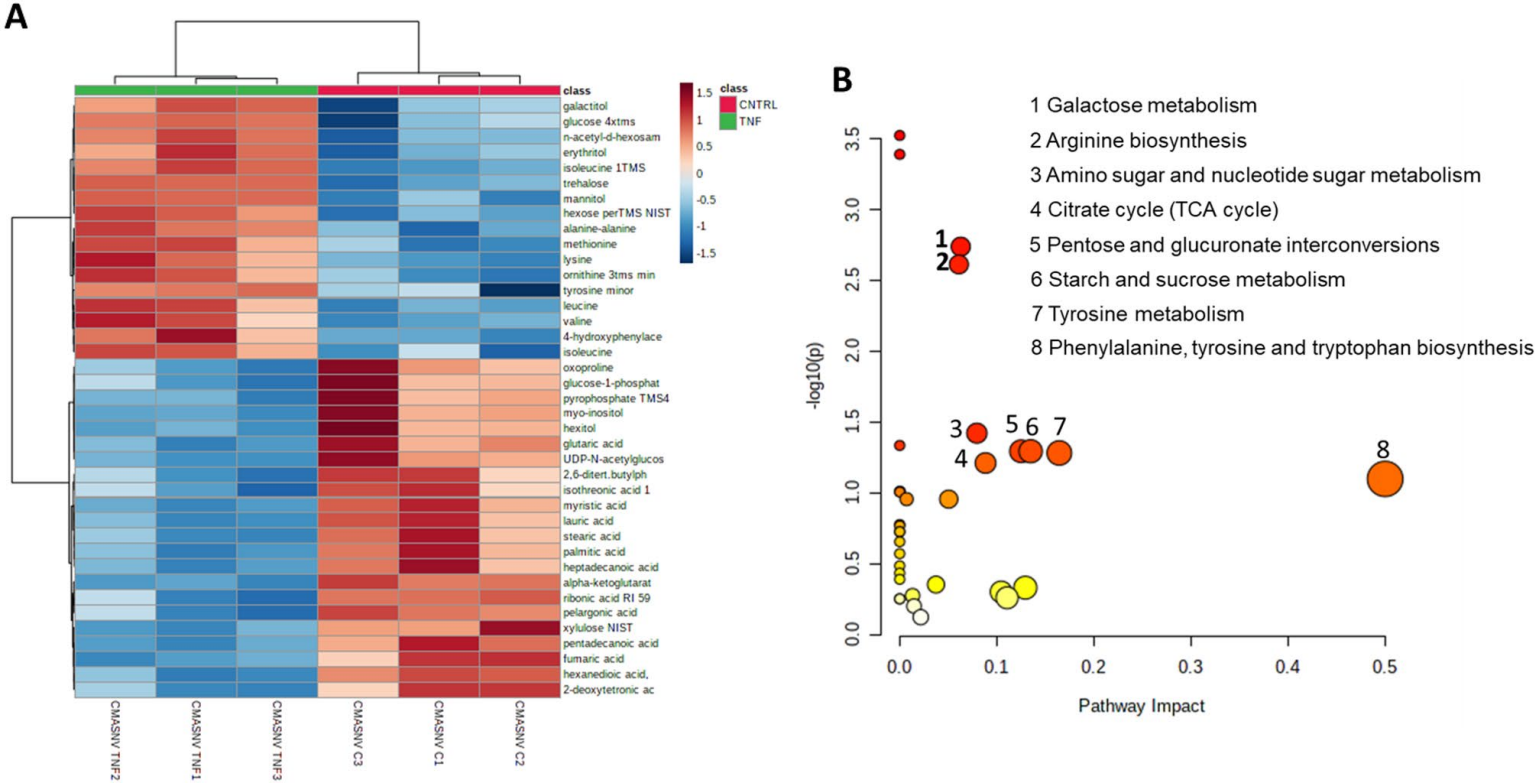
Metabolic changes in bFLS induced by bTNF-α. (**A**) Clustered heatmap of the metabolite profile changes induced by bTNF-α in bFLS. Rows represent specific intracellular metabolites, and columns represent the conditions. n = 3. (**B**) Overview of principal metabolic pathways induced bTNF-α in bFLS.

A total of 39 identified metabolites with the lowest *p* values and fold changes of ≥ 2 were grouped and visualized through a heatmap (Fig. 1A) to determine possible variations in the metabolite profile between the two groups. The clustering of metabolites led to a good separation between control bFLS and bTNF-α-treated bFLS**.**

The principal component analysis (PCA) showed an evident separation between the control group and bTNF-α-treated cells, demonstrated by Axes 1 and 2, with values of 59.5% and 18.7%, respectively (data not shown).

A total of 17 metabolites showed a significant change between both groups. The chemical classification of these metabolites includes mainly fatty acids, organic acids, amino acids and carbohydrates. We observed that glucose was higher in the bTNF-α-stimulated group than in the control group (Table 1). In contrast, the intermediates of the TCA cycle, malate, fumarate, and α-KG, were reduced by bTNF-α. Also, we observed a significant decrease in FAMEs, including stearate, palmitate, and laurate, in bTNF-α-treated bFLS. (Table 1). An increase in the amino acids isoleucine, lysine, tyrosine, leucine, and valine and a reduction in ornithine and methionine were observed in the bFLS treated with bTNF-α (Table 1). Overall, these results show that bTNF-α produces a change in the bFLS metabolome, especially in intermediate metabolites in metabolic pathways such as the TCA cycle and starch, galactose, and amino acid metabolism (Fig. 1B).

**Table 1:** List of metabolites of bFLS treated with bTNF-α.

Compound	HMDB	ChEBI	KEGG	CLASS	FC	log2(FC)	p value	(−)LOG10(p)
l-Isoleucine	HMDB0000172	17,191	C00407	Aminoacids	1258.00	10.30	0.000746	3.13
Alanyl-Alanine	HMDB0028680	72,816	C00993	Aminoacids	314.65	8.30	0.00059	3.23
l-Lysine	HMDB0000182	18,019	C00047	Aminoacids	180.45	7.50	0.036158	1.44
l-Tyrosine	HMDB0000158	17,895	C00082	Aminoacids	69.34	6.12	0.001864	2.73
l-Leucine	HMDB0000687	15,603	C00123	Aminoacids	66.38	6.05	0.004657	2.33
l-Valine	HMDB0000883	16,414	C00183	Aminoacids	34.74	5.12	0.003187	2.50
Ornithine	HMDB0000214	15,729	C00077	Aminoacids	0.34	−1.54	0.004511	2.35
l-Methionine	HMDB0000696	16,643	C00073	Aminoacids	0.33	−1.61	0.013175	1.88
p-Hydroxyphenylacetic acid	HMDB0000020	18,101	C00642	Benzenoids	9.95	3.31	0.044455	1.35
myo-Inositol	HMDB0000211	17,268	C00137	Benzenoids	7.17	2.84	0.005754	2.24
Mannitol	HMDB0000765	16,899	C00392	Carbohydrate	4.28	2.10	0.02334	1.63
Trehalose	HMDB0000975	16,551	C01083	Carbohydrate	3.50	1.81	0.011514	1.94
d-Glucose	HMDB0000122	17,634	C00221	Carbohydrate	2.51	1.33	0.011258	1.95
Galactitol	HMDB0000107	16,813	C01697	Carbohydrate	2.42	1.28	0.003507	2.46
d-Galactose	HMDB0000143	28,061	C00984	Carbohydrate	2.23	1.16	0.001464	2.83
Ribonic acid	HMDB0000867	21,077	C01685	Carbohydrate	2.19	1.13	0.00485	2.31
Threonic acid	HMDB0000943	49,059	C01620	Carbohydrate	2.11	1.08	0.007418	2.13
Galactose 1-phosphate	HMDB0000645	17,973	C00446	Carbohydrate	0.48	−1.04	0.013611	1.87
d-Xylulose	HMDB0001644	17,140	C00310	Carbohydrate	0.47	−1.08	0.007372	2.13
Galactitol	HMDB0000107	16,813	C01697	Carbohydrate	0.47	−1.08	0.004939	2.31
Stearic acid	HMDB0000827	28,842	C01530	Fatty Acids	0.44	−1.18	0.035949	1.44
Palmitic acid	HMDB0000220	15,756	C00249	Fatty Acids	0.41	−1.28	0.002008	2.70
Myristic acid	HMDB0000806	28,875	C06424	Fatty Acids	0.40	−1.31	0.004815	2.32
Diethylhexyl adipate	HMDB0040270	34,675	C14240	Fatty Acids	0.39	−1.37	0.000851	3.07
Pelargonic acid	HMDB0000847	29,019	C01601	Fatty Acids	0.39	−1.37	0.000139	3.86
Glutaric acid	HMDB0000661	17,859	C00489	Fatty Acids	0.38	−1.40	0.019703	1.71
(S)-3,4-Dihydroxybutyric acid	HMDB0000337	86,371	NA	Fatty Acids	0.36	−1.46	0.027937	1.55
Hypoxanthine	HMDB0000157	17,368	C00262	Nucleic acids	0.36	−1.48	0.005523	2.26
Uridine diphosphate-N-acetylglucosamine	HMDB0000290	16,264	C00043	Nucleic acids	0.36	−1.49	0.00751	2.12
Malic acid	HMDB0000744	6650	C03668	Organic acids	0.31	−1.68	0.038308	1.42
Oxoglutaric acid	HMDB0000208	30,915	C00026	Organic acids	0.31	−1.69	0.005798	2.24
Fumaric acid	HMDB0000134	18,012	C00122	Organic acids	0.31	−1.71	0.005489	2.26
Ethanolamine	HMDB0000149	16,000	C00189	Organic nitrogen compounds	0.30	−1.75	0.0052	2.28
Methylamine	HMDB0000164	16,830	C00218	Organic nitrogen compounds	0.25	−2.02	0.035682	1.45
Pyroglutamic acid	HMDB0000267	18,183	C01879	Organoheterocyclic compounds	0.13	−2.96	0.010247	1.99

Fold of change (FC) described are the ratio of bTNF-α/vehicle. The class, FC, log2(FC), p value, and (−)LOG10(p) were obtained from MetaboAnalyst v5.0.

### bTNF-α reduced the metabolic flux of glucose carbons into the TCA cycle and increase the accumulation of pyruvate in bFLS

**Figure 2:**
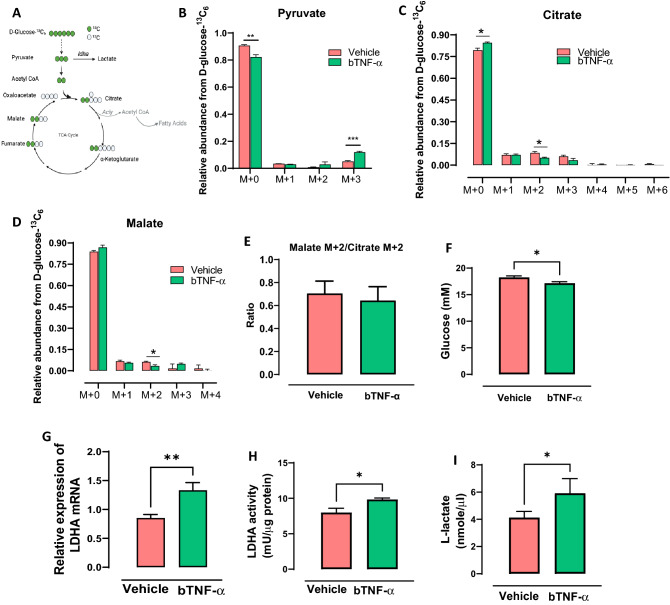
Metabolic shift induced by 100 ng/ml bTNF-α in bFLS. (**A**) Schematic representation of flux from D-glucose-^13^C_6_ metabolism. Created with BioRender.com. MID profiles of (**B**) pyruvate, (**C**) citrate and (**D**) malate derivatives from D-glucose-^13^C_6_ metabolism induced by bTNF-α. n = 3. * *p* < 0.05 compared with vehicle. (**E**) Ratio of malate M + 2/citrate M + 2 to infer fractional canonical TCA cycle. Levels of (**F**) Glucose (**G**) mRNA expression of LDHA, (**H**) enzymatic activity of LDHA and (**I**) extracellular concentration of L-lactate. n = 4. **p < 0.01; *p < 0.05 compared with the control. Each bar represents the mean ± standard error of the mean (SEM), n = 4, independent experiments.

Since we observed an increase in glucose and a reduction in TCA metabolites in bTNF-α-treated bFLS, we performed a metabolic flux assay. To monitor intracellular glucose-derived fluxes in cells, we incubated bFLS for 12 h in the presence of D-glucose-^13^C_6_ to reach isotopic steady state conditions^28^. During these periods of time, labeled glucose is metabolized inside the cells and incorporated into cellular metabolites downstream of glucose. To obtain the specific labeling patterns, intracellular metabolites were extracted and analyzed using GC/MS to determine multiple ion detections (MIDs) (corrected for natural isotope abundance), which reflect relative metabolic fluxes^28^. We observed an increase of pyruvate M + 3 and a decrease in citrate and malate M + 2 isotopologs in bTNF-α-treated bFLS, suggesting a decreased flow of glucose carbons into the TCA cycle and metabolic rewiring (Fig. 2A–D). We observed an increased abundance of citrate M+0 isotopologs, representing citrate molecules derived from carbon sources other than glucose. The M+2 abundance of malate was similar to the M+2 abundance of citrate, suggesting that either is used for the TCA cycle (Fig. 2C-E). Besides, a reduction of extracellular glucose in the conditioned medium was observed (Fig. 2F).

We have shown that bTNF-α induces a metabolic change in bFLS by significantly increasing glycolysis components and decreasing some components of the TCA cycle, which suggests that the inflammatory effect of bTNF-α on bFLS is glucose metabolism-dependent. To determine whether bTNF-α induces glycolysis reprogramming, we determined the effect of bTNF-α on the expression of LDHA in b-FLS. The results showed that bTNF-α increased the expression and activity of LDHA at 6 h of stimulation (Fig. 2G,H). LDHA is involved in the conversion of pyruvate into lactate and therefore could be accumulated in the extracellular space. In fact, we observed an increase in L-lactate at 6 h after stimulation with bTNF-α in bFLS (Fig. 2I). These results suggest that bTNF-α increases L-lactate production by modulating LDHA activity, suggesting a connection between metabolic reprogramming and the bTNF-α-induced inflammatory response in bFLS.

### Inhibition of glycolysis decreases the expression of IL-6, IL-8, IL-1β and COX-2 induced by bTNF-α in bFLS

**Figure 3:**
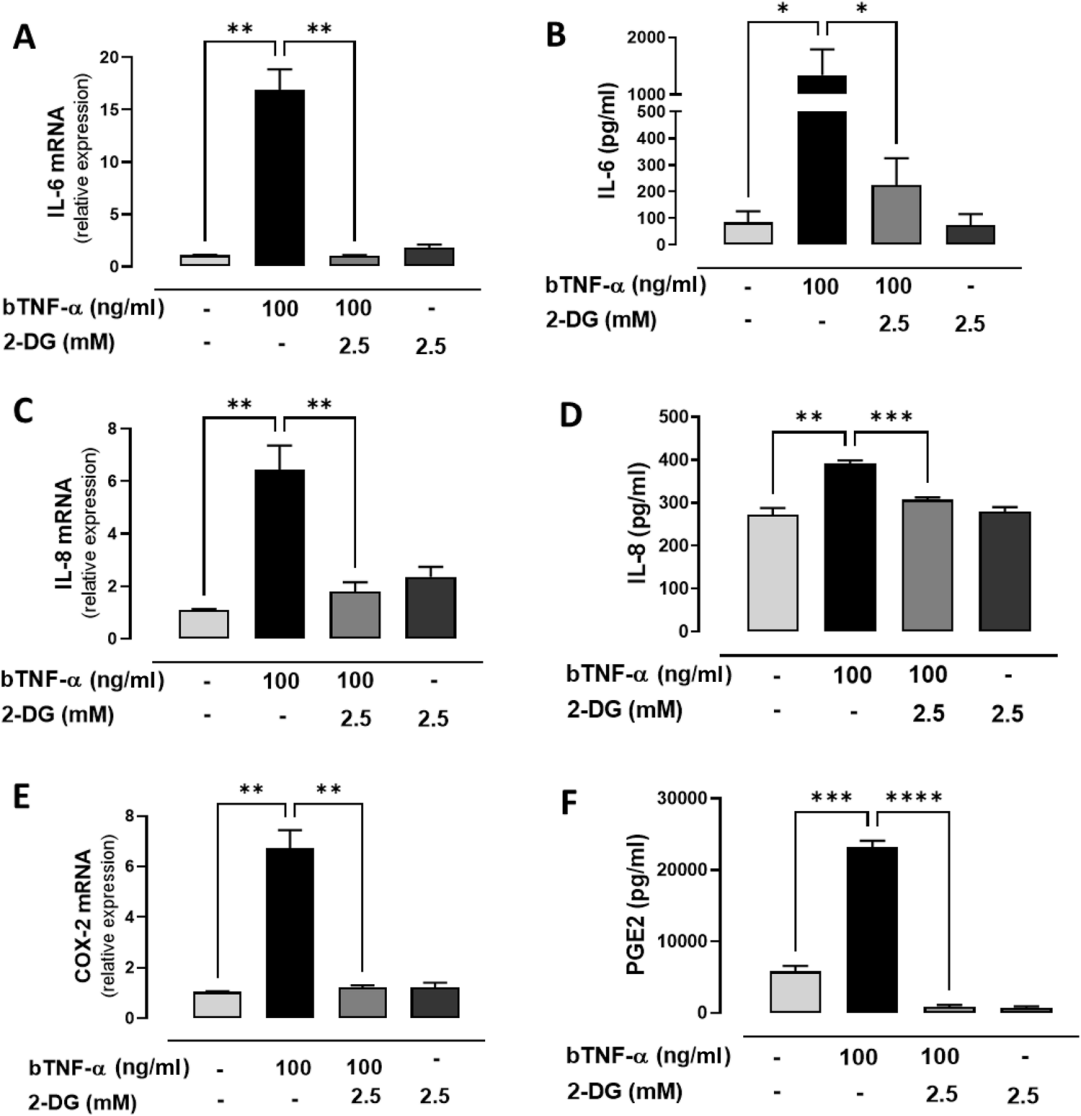
Participation of glycolysis in the expression of IL-6, IL-8 and COX-2/PGE2 in bFLS induced by bTNF-α. mRNA expression and production of IL-6 (**A**,**B**), IL-8 (**C**,**D**), and COX-2 (**E**)/PGE2 (**F**) in bFLS treated or not with 2-DG and stimulated or not with bTNF-α. ****p < 0.0001; ***0.001; **p < 0.01, and *p < 0.05 with respect to bTNF-α. Each bar represents the mean ± SEM, n = 4 independent experiments.

We have previously reported that bTNF-α increases the expression of glycolytic genes such as GLUT1 and PDK1^24^. Here, we observed that bTNF-α increases glucose, which leads us to hypothesize that bTNF-α modifies cellular metabolism toward a glycolytic state to induce proinflammatory effects. To evaluate the contribution of glycolysis to proinflammatory cytokine and COX-2 expression induced by bTNF-α, a nonhydrolyzable glucose analog, 2-Deoxy-D-glucose (2-DG), was used. 2-DG is taken up through glucose transporters and phosphorylated by hexokinase to form the nonmetabolizable intermediate 2-DG-6 phosphate (2-DG-6-P), thus interfering with glycolysis. Stimulation of bFLS with bTNF-α (100 ng/ml) for 6 h resulted in increased of expression and synthesis of IL-6 and IL-8 (Fig. 3A–D). On the other hand, bTNF-α only increased the expression, but not the secretion of IL-1β (Fig. Suppl. S2A–E). Similarly, other authors showed that in human FLS, TNF-α does not increase IL-1β secretion^29^. Hence, these results suggest that an additional stimulus might be required for inflammasome activation and subsequent processing and secretion of IL-1β. Similarly, bTNF-α treatment increased COX-2 mRNA expression and PGE2 production (Fig. 3E,F). Pretreatment with 2-DG significantly inhibited the inflammatory-related genes induced by bTNF-α (Fig. 3A–F; Fig. Suppl. S2A). These results suggest that the effects of bTNF-α on the expression of inflammatory related genes in bFLS are dependent on glycolysis.

### bTNF-α increases LDHA expression through the PI3K/Akt pathway

**Figure 4:**
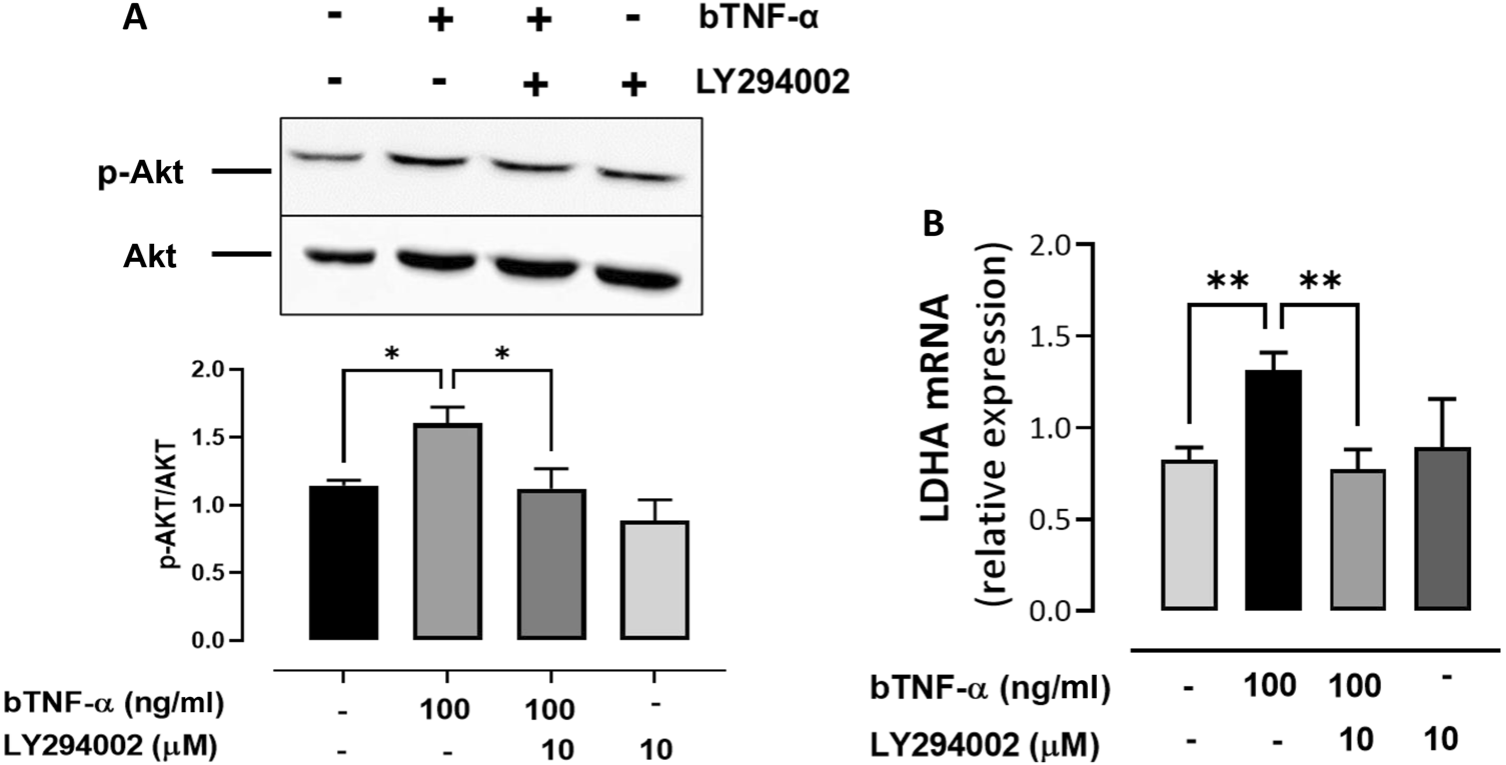
The expression of LDHA induced by bTNF-α is PI3K/Akt dependent in bFLS. (**A**) Representative image of Western blot (upper) of 4 independent experiments of p-Akt/Akt total from bFLS treated or not with LY294002 and stimulated or not with bTNF-α. (bottom) Densitometry of Western blot of p-Akt/Akt total. (**B**) Relative expression of LDHA mRNA in bFLS treated with LY294002 or not and stimulated with bTNF-α. n = 4. **p < 0.01; *p < 0.05 compared with bTNF-α treatment.

**Figure 5:**
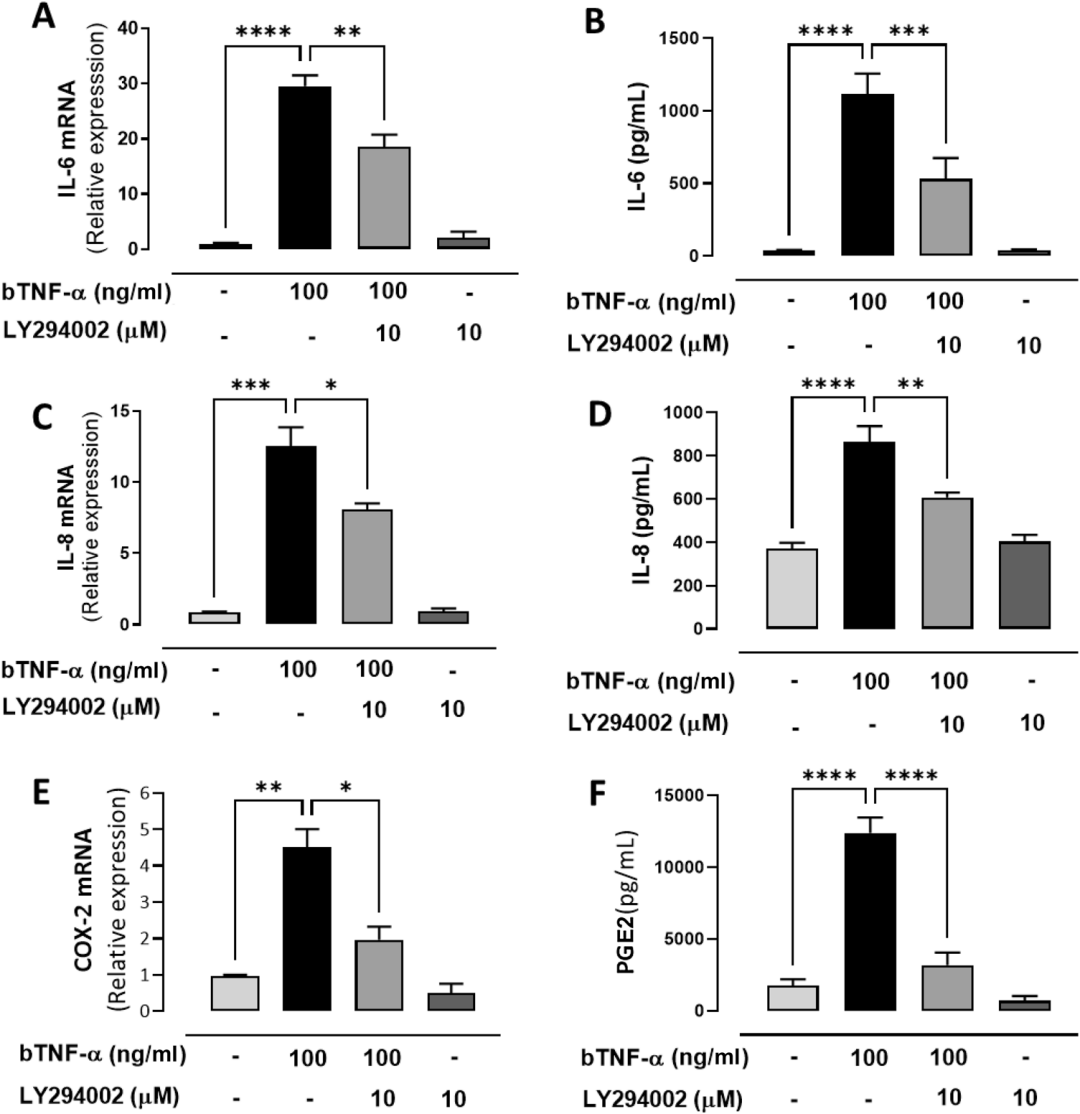
The PI3K/Akt pathway is involved in the expression of IL-6, IL-8 and COX-2/PGE2 in bFLS induced by bTNF-α. mRNA expression and production of IL-6 (**A**,**B**), IL-8 (**C**,**D**), and COX-2 (**E**)/PGE2 (**F**) in bFLS treated or not with LY294002 and stimulated or not with bTNF-α. ****p < 0.0001; ***0.001; **p < 0.01, and *p < 0.05 with respect to bTNF-α. Each bar represents the mean ± SEM, n = 4 independent experiments.

The PI3K/Akt signaling pathway is closely associated with a variety of enzymatic biological effects and glucose metabolism^30^. Here, we demonstrate that bTNF-α significantly increased Akt phosphorylation at 5 min of stimulation and decreased Akt phosphorylation when the PI3K inhibitor LY294002 was used (Fig. 4A). In addition, we demonstrate that the inhibitor LY294002 reduces the expression of LDHA (Fig. 4B), suggesting that the increase in LDHA expression induced by TNF-α is dependent on the PI3K/Akt signaling pathway. The PI3K/Akt pathway plays an important role in synovial inflammation^31^; in fact, we demonstrated that a PI3K inhibitor partially decreased the bTNF-α-induced expression of IL-6, IL-8, IL-1β and COX-2 (Fig. 5A,C,E; Fig. Suppl. S2B). Additionally, LY294002 significantly decreased the secretion of IL-6, IL-8 and PGE2 (Fig. 5B,D,F). These results suggest that the PI3K/Akt-LDHA pathway might be involved in the inflammatory response and that TNF-α-induced metabolic shift in bFLS.

### Inhibition of LDHA decreases the inflammatory response induced by bTNF-α in bFLS

**Figure 6:**
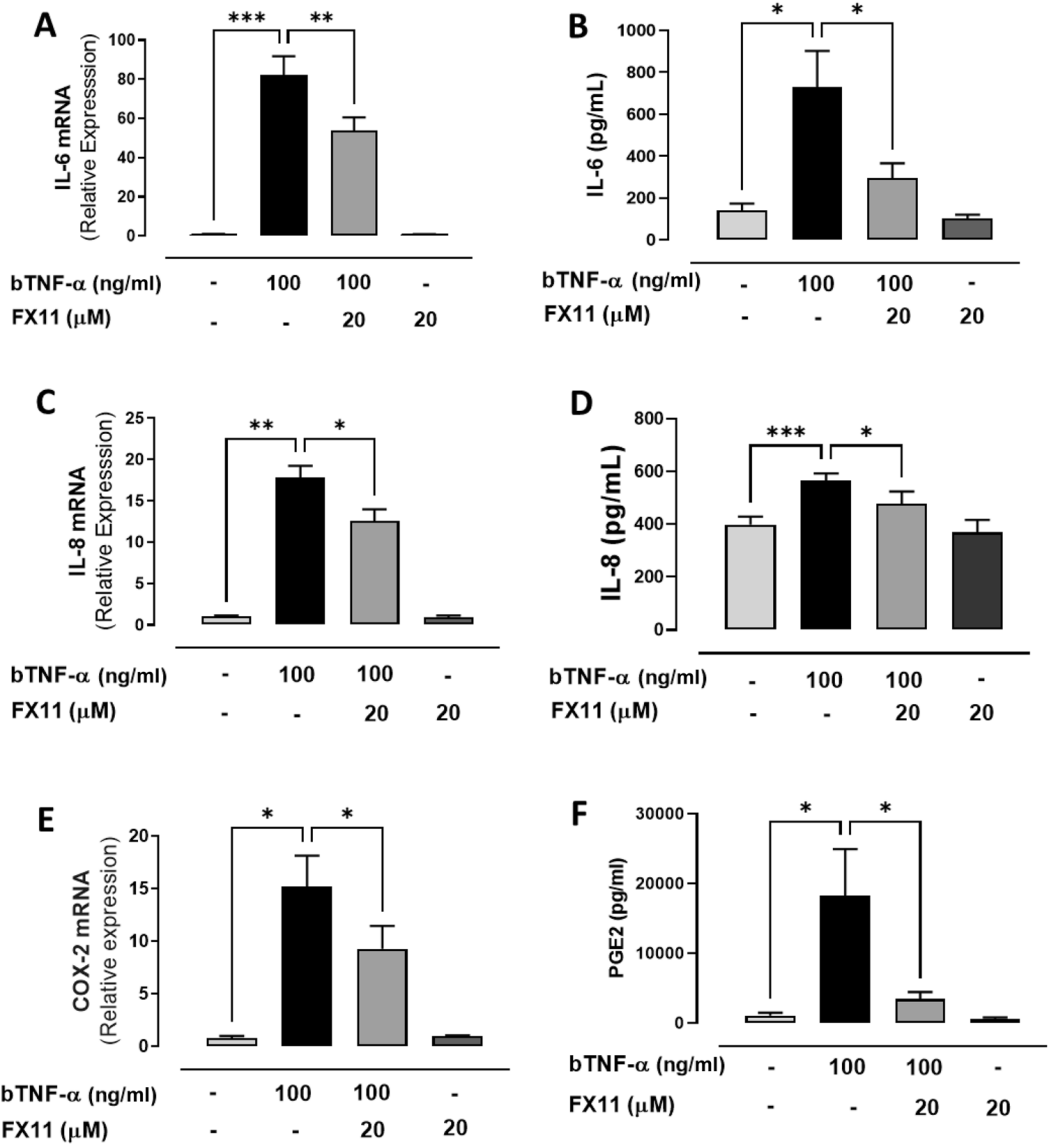
Involvement of LDHA in the expression of IL-6, IL-8 and COX-2/PGE2 in bFLS induced by bTNF-α. mRNA expression and production of IL-6 (**A**,**B**), IL-8 (**C**,**D**), and COX-2 (**E**)/PGE2 (**F**) in bFLS treated or not with LY294002 and stimulated or not with bTNF-α. ***p < 0.001; **p < 0.01, and *p < 0.05 with respect to bTNF-α. Each bar represents the mean ± SEM, n = 4 independent experiments.

As previously demonstrated, bTNF-α increases the expression of LDHA, an enzyme that converts pyruvate to lactate with the production of NAD^+^. Here, we evaluated whether the inhibition of LDHA by FX11 has anti-inflammatory effects on bFLS stimulated with bTNF-α. We observed a partial decrease in the expression and secretion of IL-6 and IL-8 (*p* < 0.05). Additionally, COX-2 mRNA expression and PGE2 synthesis induced by bTNF-α decreased in the presence of FX11 (p < 0.05) (Fig. 6A–F). In addition, FX11 decreased the IL-1β expression in bFLS (Fig. Suppl. S2C). In contrast, dichloroacetate (DCA), a pyruvate dehydrogenase kinase inhibitor, only reduced IL-6 expression but did not affect IL-8, IL-1β and COX-2 expression induced by bTNF-α in bFLS (Fig. Suppl. S1A–C; Fig. Suppl. S2D). These results suggest that bTNF-α induces a metabolic change in bFLS, shifting from OXPHOS to aerobic glycolysis, with an increase in lactate levels, which could contribute in part to the inflammatory effect mediated by bTNF-α.

## Discussion

Lameness is one of the most important conditions that affect health and welfare in the dairy industry, causing enormous economic losses worldwide. Arthritis-synovitis is a condition associated with lameness in cattle^11,32^, and TNF-α is a proinflammatory cytokine responsible for the development of the inflammatory process and tissue damage^33,34^.

FLS are predominant cells of the synovium and contribute to inflammatory and metabolic changes during joint diseases^22,35^. RA-FLS are more glycolytic than FLS from healthy patients^36^. In RA-FLS, TNF-α increases the production of proinflammatory cytokines and glycolysis^29^; moreover, the use of antiTNF antibody downregulates the induced glycolysis and rescues mitochondrial function^37,38^. In accordance, antiTNF therapy has been shown to decrease glucose uptake and reduce the inflammatory response in RA joints^37,39^. We observed that the intracellular level of glucose was higher in bFLS treated with bTNF-α. In fact, we observed an increase of metabolic flux by using D-glucose-^13^C_6_, which produced pyruvate containing three heavy-labeled carbons (M + 3-labeled pyruvate) and decreased of extracellular glucose level in the conditionate medium. In contrast, the TCA cycle intermediates malate, fumarate, and α-KG were reduced. These results correlate with previously published data, which have shown that stimulation of bFLS by bTNF-α increases the expression of HIF-1α, GLUT1 and PDK1, which favors a glycolytic metabolic phenotype-like^29^. We have previously shown that lactate exert proinflammatory effects in bFLS^40^. Lactate increases in the joint of animals with aseptic polysinovisits, inducing a metabolic reprogramming of FLS and increasing the expression of glycolytic enzymes and pro-inflammatory molecules like TNF-α^24^. Other authors, using co-culture of CD4 + T cells, demonstrated a similar effect on FLS, suggesting that the secretion of proinflammatory products of CD4 + T can promote glycolysis in FLS while downregulating OXPHOS^41^.

In a rat model of arthritis, synovial pannus cells increase glucose uptake^42^, and HK2, a rate-limiting enzyme of glycolysis, regulates the aggressive functions of FLS^21^. Furthermore, the interference of glycolysis via HK2 inhibition ameliorates arthritis in an animal model of inflammatory arthritis^21^. In our study, inhibition of glycolysis with 2-DG reduced the expression of proinflammatory genes in bFLS stimulated with bTNF-α. Treatment with 2-DG has been shown to inhibit IL-6 in RA-FLS^29^. Moreover, 5 mg/kg 3-bromopyruvate (glycolysis inhibitor) in a mouse with arthritis induced by K/BxN mouse serum reduces clinical score, proinflammatory cytokines in the joint, inflammatory cell infiltration, joint destruction, and cartilage damage^27^, which has made it possible to consider glycolysis pathway inhibition as a potential pharmacological target for RA^29^.

**Figure 7:**
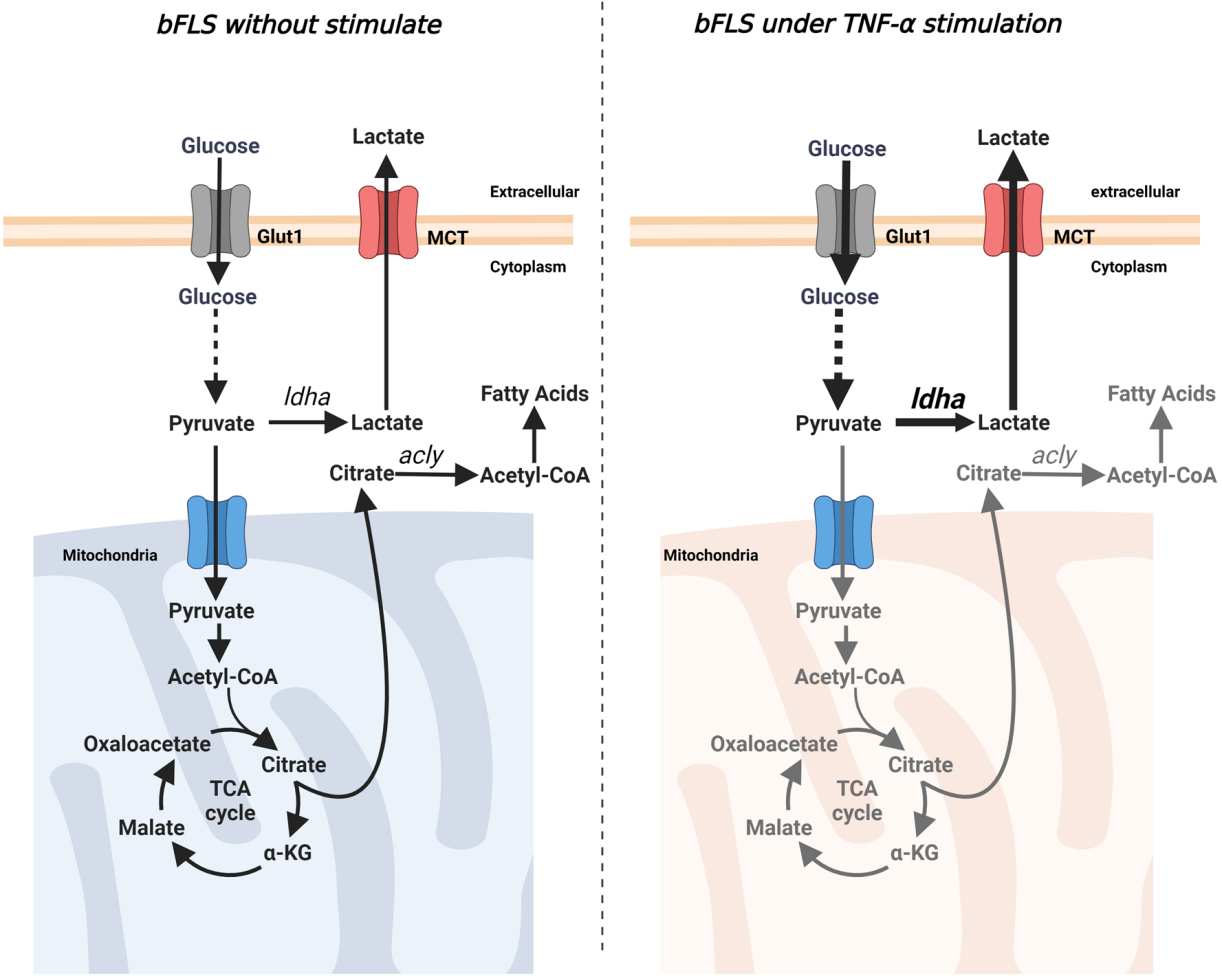
bTNF-α induces metabolic rewiring that is closely related to the proinflammatory phenotype of bFLS. bTNF-α increases the entry of glucose and glycolysis and disrupts the TCA cycle, increasing LDHA activity and lactate extracellular concentration via monocarboxylate transporters (MCT). In addition, bTNF-α reduces fatty acid synthesis via citrate/acetyl-CoA. *acly*: ATP-dependent citrate lyase Created with BioRender.com.

We observed a reduction in several FAMEs, such as stearate, palmitate, and myristate, induced by bTNF-α. Similarity in RA-FLS, a reduction in fatty acids induced by TNF-α has been observed^27^. The decrease in citrate levels could explain the fatty acid changes since citrate is required for the synthesis of fatty acids via acetyl-CoA^28^. To assess the metabolic changes induced by bTNF-α in bFLS, we performed a metabolic flux analysis by using d-glucose-^13^C_6_, which generates citrate containing two heavy-labeled carbons (M + 2-labeled citrate) following oxidative decarboxylation of glucose-derived pyruvate. Our results confirm a decrease in M + 2 isotopologs of citrate and therefore a reduction in glucose carbon in the TCA cycle (Fig. 7). Additionally, we observed a decrease in M + 2-labeled malate, suggesting that M + 2-labeled citrate is properly metabolized by aconitase in the traditional TCA cycle, generating M + 2-labeled malate; thus, the reduction in fatty acids in bFLS treated with bTNF-α could be attributable to the reduction in the noncanonical TCA cycle via ATP-dependent citrate lyase (ACLY) and acetyl-CoA^43^. Acetyl-CoA is the precursor for fatty acid synthesis in the cytoplasm. Acetyl-CoA is produced from pyruvate via pyruvate dehydrogenase (PDH); however, its activity could be reduced since the increase in PDK1 expression observed in bFLS treated with bTNF-α^24^, reducing the flux of glucose carbon to the TCA cycle observed in cells treated with TNF-α. A reduction in the conversion of pyruvate to acetyl-CoA may also affect fatty acid synthesis while also driving pyruvate conversion to lactate, favoring glycolysis^44^(Fig. 7).

In human FLS, TNF-α increases glycolysis and slightly oxygen consumption in a dose-dependent manner, suggesting that the contribution of mitochondria to energy production is limited^29^. In RA-FLS, an increase in glutamine transporters could support the role of glutamine as an alternate carbon source in the absence of glucose^45^. We observed that in bTNF-α-treated bFLS, the oxidative TCA cycle is still running and could be fueled by glutamine in the culture medium via α-KG.

The reduction in glucose flux toward the TCA cycle favors rewiring to lactate production. In fact, we observed that bTNF-α increases extracellular L-lactate in bFLS. LDHA actively reduces pyruvate to lactate and is considered a master regulator of aerobic glycolysis^46^. Previously, we demonstrated that bTNF-α increases the expression of GLUT1 and LDHA in bFLS^24^. Our results showed that bTNF-α induces an increase in the expression and activity of LDHA. In addition, in inflammatory processes, acidification of the medium occurs due to the increase in lactate secretion through LDHA-mediated fermentation^47^. Associated with this increase, a decrease in a set of OXPHOS genes has been observed during inflammation^47^.

Lactate levels are known to increase with the degree of inflammation in the joint^40^. The physiological concentration of lactate in healthy individuals is in the range of 1.5–3 mM. However, lactate levels can increase up to 10 mM in inflammatory pathologies (for example, rheumatic synovial fluid)^48^. Lactate accumulation in synovial fluid is caused by the high metabolic demand of synovial cells in RA patients^48^. In addition, a positive correlation between LDHA and high lactate levels is observed in the serum and synovial fluid of RA patients^48^. However, there is little evidence linking the effect of TNF-α on LDHA activity and increased lactate in inflammatory processes^46^. In agreement with this, we showed that inhibition of LDHA with FX11 reduced the expression and synthesis of proinflammatory cytokines induced by bTNF-α. Similarly, FX11 reduces the expression of IL-6, IL-1β, TNF-α, iNOS and COX-2 in LPS-treated RAW 264.7 cells^49^. LDHA inhibition is considered a potential target to reduce deleterious inflammatory and cytolytic contributions of blood CD8 + T cells in RA patients^50^. Moreover, in vitro experiments have demonstrated that miR-34a-5p downregulates the increase in LDHA expression induced by the long noncoding RNA TUG1 in RA-FLS^51^. Altogether, these findings suggest that LDHA and/or lactate are common elements at the crossroads between cell metabolism and inflammation^46^.

We observed that the expression of LDHA induced by bTNF-α was dependent on the PI3K/Akt signaling pathway in bFLS. In lymphocytes, PI3K-dependent phosphorylation of Akt has been shown to exhibit a response that is directly proportional to LDHA, since inhibition of PI3K signaling repressed the expression of LDHA and its transcriptional regulator c-Myc^52^. In addition, PI3K/Akt signaling promotes the expression of the glucose transporter GLUT1, which shows that PI3K regulates glucose metabolism in lymphocytes (Teff cells)^52^. Recently, we demonstrated that the PI3K/Akt pathway controls the expression of the HIF-1α subunit induced by bTNF-α in bFLS^24^. Besides, we previously demonstrated that TNF-α increases p-38 MAPK phosphorylation, NF-κB and HIF-1 pathway, suggesting that additional cellular signaling is required for IL-6, IL-8, and COX-2 expression in bFLS^20,24^. HIF-1 is a transcription factor that controls several genes required to maintain a balance between oxygen supply and metabolic demand^53^. In addition, TNF-α induces the expression of PDK1 via PI3K/Akt-HIF-1 in bFLS^24^ and thus might interfere with pyruvate mitochondrial utilization through the TCA cycle^53^. Inhibition of PDKs can reprogram metabolism from glycolysis toward OXPHOS, reducing inflammatory cytokine secretion^54^. DCA has been described as an allosteric PDK inhibitor that, at mM concentration (IC50) and in concomitance with ADP, binds to the pyruvate pocket of the PDK complex enzyme, avoiding attachment of the kinase domain to the E2 domain^55,56^. DCA only reduced the expression of IL-6, suggesting that PDK1 is partially involved in the proinflammatory effects of bTNF-α in bFLS. Supporting the above, DCA can delay the onset and reduce the progression of murine collagen type II (CII)-induced arthritis^57^ and moderate the procatabolic effect of IL-1β in bovine chondrocytes^58^.

Bovine lameness is a common pathology that is closely related to laminitis and arthritis^1^. At present, no analgesic drugs, including NSAIDs, are approved to alleviate lameness-associated pain in lactating dairy cattle in USA^59^. In the present work, we demonstrated that an increase in glycolysis in bFLS is critical for the proinflammatory response induced by TNF-α and suggests the existence of new potential pharmacological targets^24,40^ that could be useful for the control of inflammatory processes in the locomotor system of cattle.

## Methods

### Cell culture

The isolation of bovine FLS (bFLS) was performed as described by Manosalva et al.^20^. Briefly, bFLS were isolated from the carpometacarpal joint without injuries to five Black Friesian multiparous dairy cows, 400–500 kg body weight (b.w.), obtained from a local slaughterhouse. Experiments were conducted in accordance with the recommendations of Agencia Nacional de Investigación y Desarrollo based on Chilean Animal Protection Laws. The study protocol was endorsed by the ethical committee of Universidad Austral de Chile (0023/18; Valdivia, Chile). The synovial membrane was digested with 0.2% type IV collagenase (Thermo Fisher Scientific, Waltham, MA, USA) for 2 h at 37 °C with gentle shaking. The suspension cells were seeded in Dulbecco’s modified Eagle’s medium/Nutrient Mixture F12 (DMEM/F12; #12400016, Gibco, Thermo Fisher Scientific) containing 10% fetal bovine serum (FBS; Biowest, Nuaillé, France) in a 75-cm^2^ flask. The cells were incubated with 5% CO_2_ at 37 °C, and passages 2–6 were used for the experiments. To confirm the identity of bFLS, CD14 (#557742. Becton Dickinson, Franklin Lakes, NJ, USA) and Vimentin (#BM5501F. Acris, Herford, Germany) antibodies were used, as described previously^20^.

### Sample preparation for metabolomics analysis

For metabolomic analysis, bFLS were stimulated with 100 ng/ml bovine TNF-α (bTNF-α. #RBOTNFAI, Thermo Fisher Scientific, Waltham, MA, USA) at 37 °C and under 5% CO_2_ for 1 h in sterile 21.5-cm^2^ plastic tissue culture plates (SPL Life Sciences, Pocheon-si, Korea). To obtain the metabolome samples, bFLS were washed twice with 0.9% NaCl and then frozen for 3 min in liquid nitrogen. Next, 1 ml extraction buffer (37.5% vol/vol liquid chromatography/mass spectrometry (LC/MS) grade acetonitrile; 37.5% vol/vol LC/MS-grade isopropanol; 25% vol/vol LC/MS-grade water) containing 1 mM ribitol (# A5502, Sigma–Aldrich, St. Louis, MO, USA) was added to the cells. For the detachment and collection of the samples, a rubber-tipped cell scraper was used. The samples were vortexed for 2 min and then centrifuged at 14,000×*g* at 4 °C for 2 min. Then, 450 µl of supernatant was collected and completely dried under vacuum using a SpeedVac vacuum concentrator (Savant® SPD131DDA, Thermo Fisher Scientific, Waltham, MA, USA) at 45 °C for 90 min at 1.5 atm pressure. Dried samples were treated with 450 μl of wash buffer (50% vol/vol LC/MS-grade acetonitrile; 50% vol/vol LC/MS-grade water) and vortexed for 3 min. After centrifugation at 14,000×*g* at 4 °C for 2 min, the supernatants were evaporated to dryness in a SpeedVac concentrator at 45 °C for 90 min at 1.5 atm pressure. The dried samples were derivatized with 10 μl methoxyamine/pyridine hydrochloride (20 mg/ml; #226904, Sigma–Aldrich, #107463, Merck KGaA, Darmstadt, Germany) at 30 °C for 90 min. Then, 90 μl of MSTFA with 1% TMCS derivatization agent (#TS-48915, Thermo Fisher Scientific, Pierce Biotechnology, Rockford, IL) was added in concomitance with 2 μl of a mixture of C8-C30 FAMEs (# 400505, Fiehn GC/MS Metabolomics Standards Kit, Agilent Technologies, Santa Clara, CA, USA), used as markers of retention times and incubated at 37 °C for 30 min under shaking conditions. Finally, the samples were transferred to 250-μl glass vial inserts (#5181-8872, Agilent Technologies) in 2-ml glass vials with screw caps (#8010-0543, Agilent Technologies) for analysis.

### Untargeted metabolomics analysis by GC/MS

For sample analysis, an Agilent 7890B GC system coupled to an electron ionization (EI) mode 5977 A mass selective detector (Agilent Technologies) was used. Briefly, 1 μl of derivatized sample was injected in splitless injector mode into an integrated guard column (J&W DB-5 ms GC Column, 30 m, 0.25 mm, 0.25 µm, DuraGuard, 10 m; #122-5532G, Agilent Technologies) for the separation of metabolites in the samples. The injector port temperature was held at 250 °C, and the helium carrier gas flow rate was set to 1 ml/min at an initial oven temperature of 60 °C. Then, the oven temperature was increased to 325 °C at a rate of 10 °C/min for a final run time of 37.5 min. The mass spectra were collected in the mass range of 850–600 m/z at an acquisition rate of 1.7 spectra/s with a digital scan rate of 20 Hz and were acquired after a 5.9 min solvent delay, with an MS ion source temperature of 250 °C and quadrupole temperature of 150 °C.

Metabolite identification was performed according to the methods described by Fiehn. Briefly, peak detection, deconvolution and peak alignment in data processing were performed using MSDIAL 2.83 (RIKEN Center for Sustainable Resource Science: Metabolome Informatics Research Team. Yokohama, Japan). Then, for deconvolution, each of the trimethylsilylated metabolites were identified, and deconvoluted peaks were matched against mass spectral libraries that were imported into the NIST MSP format. Library match hits were ranked against experimental data based on the total retention index and mass spectral similarity across all samples that were processed in a batch. The retention index, Fiehn RI, based on FAME, was used. Metabolites were identified by matching the EI spectra with those of reference compounds from the NIST or Fiehn libraries. For the analysis, we used a 2000 retention index tolerance, 70% EI similarity cutoff, 70% identification score cutoff, 0.5 Da m/z tolerance and 0.5 min retention time tolerance.

### Metabolic flux analysis

For this analysis, we used the protocol described by Saunders et al.^60^ with modifications. Cells were incubated at 70–80% confluence in 6-well plates at 37 °C and 5% CO_2_ for 3 days in DMEM/F12 with 10% FBS. Then, the medium was replaced with DMEM without glucose but with 0.5% FBS. D-Glucose-^13^C_6_ (#389374; Merck, Chile, 25 mM) was added and incubated at 37 °C and 5% CO_2_ for 12 h to reach the steady state of the isotope tracer. Next, the medium was replaced with DMEM/F12 with 10% FBS plus 25 mM D-glucose-^13^C_6_, and then the cells were treated with vehicle or 100 ng/ml bTNF-α and incubated at 37 °C and 5% CO_2_ for 1 h. As a control, cells with 25 mM glucose were not isotopically labeled. Then, the well plates were washed with 1 ml of 0.9% NaCl, and the cells were lysed with 1 ml of chloroform:MeOH:H_2_O (1:3:1) and centrifuged at 15,000×*g* for 5 min at 4 °C. Then, 450 µl of supernatant was collected and dried by using a SpeedVac concentrator. Afterward, sample derivation was performed; briefly, 20 µl of 2% methoxyamine chloride was added, and the sample was shaken for 2 h at 30 °C. Then, 20 µl BSTFA containing 1% TMCS was added, and the sample was shaken for 1 h at 30 °C. Simultaneously, a reagent blank and a standard mix that contained malate and citrate were derivatized and analyzed by GC/MS.

GC was performed using a DB5ms capillary column (J&W DB-5 ms GC Column, 30 m, 0.25 mm, 0.25 µm, DuraGuard, 10 m; #122-5532G, Agilent Technologies). The GC inlet and GC/MS transfer line temperatures were maintained at 270 °C and 250 °C, respectively. The oven temperature gradient was programmed as follows: 70 °C (1 min); 70–295 °C at 12.5 °C/min; 295–320 °C at 25 °C/min; and 320 °C for 2 min.

GC/MS data were imported into CDF-formatted files and analyzed by using DExSI for MIDs and natural isotope abundance correction^61^.

### Quantitative real-time polymerase chain reaction

bFLS were seeded in 6-well plates and treated with vehicle (H_2_O) or 100 ng/ml bTNF-α for 6 h at 37 °C and 5% CO_2_. Prior to stimulation with bTNF-α, the cells were incubated with vehicle (0.1% DMSO) or different inhibitors. Preincubation with 2.5 mM 2-deoxy-D-glucose (2-DG; #14325, Cayman Chemical, Ann Arbor, MI, USA) was performed in medium containing 5.5 mM glucose for 1 h. Pretreatment with 10 μM LY294002 (#V1201, Promega, Madison, WI, USA) and 20 μM FX11 (#HY-16214. MedChemExpress, Monmouth Junction, NJ, USA) and 25 mM sodium dichloroacetate (DCA. #sc-203275. Santa Cruz Biotechnology, Dallas, TX, USA) was performed for 30 min, 60 min and 60 min, respectively. The supernatant was collected and stored for cytokine and PGE2 detection.

Total RNA was extracted from bFLS using an RNA I kit (Omega Bio-Tek Inc., Norcross, GA, USA) according to the manufacturer's protocol. RNA was treated with DNAse to ensure removal of genomic DNA. Equal amounts of RNA (250 µg) were reverse-transcribed using M-MLV Reverse Transcriptase (Promega, Madison, WI, USA). Real-time polymerase chain reaction (PCR) was performed with Takyon™ Rox SYBR® master mix (Eurogentec, Seraing, Belgium) in a StepOnePlus™ (Applied Biosystems, Life Technologies, Foster City, CA, USA) using the following primers: IL-6 F 5ʹACTGGCAGAAAATAAGCTGAATCTTC 3ʹ and R 5ʹTGATCAAGCAAATCGCCTGAT3ʹ; IL-8 F 5ʹATGACTTCCAAGCTGGCTGTTG 3ʹ and R 5ʹTTGATAAATTTGGGGTGGAAAG 3ʹ; IL-1β F 5ʹCCTCCGACGAGTTTCTGTGT 3ʹ and R 5ʹGCCAGCACCAGGGATTTTTG 3ʹ; COX-2 F 5ʹGCATAAGCTGCGCCTTTTCA3ʹ and R 5ʹCAGGAACATGAGGCGGGTAG3ʹ; LDHA F 5ʹAGGCCTGAGAAGTCGGAGTG 3ʹ and R 5ʹGGAACCTGTCCTACCTGCC3ʹ; RPS-9 F 5ʹGCTGACGCTGGATGAGAAAGACCC3ʹ and R 5ʹATCCAGCACCCCGATACGGACG 3ʹ. The following conditions were used: 40 cycles at 95 °C for 30 s, 60 °C for 30 s (annealing), and 72 °C for 30 s (extension)^24^. The expression levels were normalized to the expression of the housekeeping gene RSP-9 (40S ribosomal protein S9) and then quantified using the 2^−ΔΔCt^ method according to Livak and Smittgen^62^ using StepOne™ v2.3 software (Applied Biosystems).

### Quantification of IL-6, IL-8, IL-1β and PGE2 by enzyme-linked immunoassay (ELISA)

Supernatants obtained from the qPCR assay were centrifuged at 600×*g* for 6 min and used to estimate concentration using bovine IL-8 ELISA kits (#3114-1A-6, Mabtech, Nacka, Sweden), IL-6 (#ESS0029, Thermo Fisher Scientific), IL-1β (#ESS0027, Thermo Fisher Scientific) and PGE2 (#514010, Cayman Chemicals, Ann Arbor, MI, USA) according to the manufacturer's instructions. Briefly, capture antibody was added to a 96-well plate and incubated overnight. The next day, the wells were blocked for 1 h (4% bovine serum albumin (BSA), 5% sucrose in phosphate-buffered saline (PBS)), and then 100 µL of sample was added and incubated for 1–2 h. After washing the plates twice, the detection antibody was added and incubated for 1 h. After two additional washes, streptavidin was added, and the mixture was incubated for an additional 0.5–1 h. Finally, *p*-nitrophenyl phosphate (pNPP) or TMB substrate solution was added, followed by incubation for 20–25 min in the dark. All procedures were performed at room temperature. The reaction was stopped with 0.16 M H_2_SO_4_ (for the IL-6 ELISA kit), and the samples were analyzed at 405, 450, 450 and 420 nm for IL-8, IL-6, IL-1β and PGE2, respectively, in a Varioskan Flash Reader (Thermo Fisher Scientific).

### Immunoblot

To measure Akt phosphorylation, confluent bFLS were treated with bTNF-α for 5 min at 37 °C under 5% CO_2_. In another set of experiments, cells were preincubated with 10 μM LY294002 for 30 min at 37 °C and then stimulated with bTNF-α for 5 min. Next, cells were lysed with lysis buffer (50 mM Tris–HCl, pH 7.4, 150 mM NaCl, 1 mM EDTA, 1 mM EGTA, 10 µg/ml aprotinin, 10 µg/ml leupeptin, 5 mM PMSF and 1 mM DTT and subsequently centrifuged at 18,000×*g* for 20 min at 4 °C. The protein concentration was determined with the Bradford reagent (Sigma-Aldrich, Saint Louis, MO, USA). Fifty micrograms of protein was resolved on 10% SDS-PAGE gels and transferred to nitrocellulose membranes. The membranes were blocked with 5% skim milk in TBS/T (20 mM Tris–HCl, pH 7.6, 137 mM NaCl and 0.05% Tween 20) and incubated with an anti-pAkt Ser 473 antibody (#9271. Cell Signaling Technology, Beverly, MA, USA) overnight. To carry out the loading control, the membranes were subjected to stripping and incubated with an antiAkt antibody (#4691S. Cell Signaling Technology). Finally, the membranes were incubated with a horseradish peroxidase (HRP)-conjugated antirabbit IgG secondary antibody (Santa Cruz, CA, USA), and bands were visualized using the Odyssey Fc Dual-Mode Imaging System (LI-COR Biosciences, Lincoln, NE, USA). Band density was measured using ImageJ 1.35 s software (Wayne Rasband, National Institutes of Health, Bethesda, MD, USA).

### Quantification of extracellular l-lactate and glucose

Supernatants from bFLS treated with bTNF-α or vehicle were harvested 6 h after stimulation. The supernatant was loaded into Amicon® Ultra4 3 kDa (#UFC800324. Merck Millipore, Darmstadt, Germany) tubes and centrifuged at 75,000×*g* for 40 min at 4 °C. l-Lactate was quantified in the filtrate with an assay kit (#MAK064, Sigma-Aldrich) via colorimetric (570 nm) measurements in a Varioskan Flash Reader using the manufacturer's protocol. The glucose level in the conditioned medium was measured using a glucose assay kit, following the manufacturer's instructions (#1400060, Wiener lab., Rosario, Argentina).

### Determination of lactate dehydrogenase (LDH) activity

bFLS were treated with bTNF-α or vehicle, and after 6 h, were homogenized on ice in 500 µl of cold lactate dehydrogenase (LDH) assay buffer. The homogenate was centrifuged at 10,000×*g* for 15 min at 4 °C to remove soluble material. LDH activity was determined in the soluble fraction using an LDH activity assay kit (#MAK066. Sigma-Aldrich) according to the manufacturer’s protocol. LDH activity was normalized based on the amount of total protein.

### Statistical analysis

For metabolomic analysis, metabolites were normalized with ribitol (internal standard), with logarithmic transformation and autoscaling to proceed with the statistical analysis. Multivariate statistical analysis was performed using MetaboAnalyst v5.0 (Xia Lab, McGill University, Montreal, Quebec, Canada; http://www.metaboanalyst.ca). The heatmap was generated with the Euclidean distance measure and Ward’s clustering algorithm. For pathway analysis, only the metabolites with significant differences were used for topology analysis using *Bos taurus.* Each experiment was performed at least three times with cells of a different passage and donor. One-way analysis of variance (ANOVA) was performed, followed by Fisher’s least significant difference (LSD) multiple comparison test was applied. When assumptions of normality or homogeneity of variance were not met according to the Shapiro-Wilks or Brown-Forsythe test, respectively, Kruskal–Wallis ANOVA and Dunn’s multiple comparison test were used. All statistical analyses were performed using GraphPad Prism v7.0 (GraphPad Software, La Jolla, CA, USA). A *p* value < 0.05 was considered significant.

## Supplementary Information

Supplementary Information 1.

Supplementary Information 2.

Supplementary Information 3.

Supplementary Information 4.

## Data Availability

The list of metabolites, normalized or not, are publicly available in the figshare repository (https://doi.org/10.6084/m9.figshare.20280363.v2). All the images summarized in the Fig. 4A are publicly available in the figshare repository, as part of this record: https://doi.org/10.6084/m9.figshare.20264502.v1. For the rest datasets from all of figures, can be accessed on upon reasonable request, to Dr. Rafael A. Burgos or Dr. John Quiroga A.

## Abbreviations

2-DG: 2-Deoxy-d-glucose
α-KG: α-Ketoglutarate
Akt: Serine/threonine protein kinase
bFLS: Bovine fibroblast-like synoviocyte
BW: Body weight
CCL-2: Chemokine (C–C motif) ligand 2
COX-2: Cyclooxygenase 2
CXCL-10: C–X–C motif chemokine ligand 10
DCA: Dichloroacetate
DExSI: Data Extraction for Stable Isotope-labeled metabolites
DMEM/F12: Dulbecco’s modified Eagle’s medium/Nutrient Mixture F12
DMSO: Dimethyl sulfoxide
DTT: Dithiothreitol
EDTA: Ethylenediaminetetraacetic acid
EGTA: Ethylene glycol-bis(β-aminoethyl ether)-N,N,Nʹ,Nʹ-tetraacetic acid
FAMEs: Fatty acid methyl esters
FBS: Fetal bovine serum
GC/MS: Gas chromatography/mass spectrometry
GLUT1: Glucose transporter 1
HIF-1: Hypoxia-inducible factor 1
HK2: Hexokinase-2
HPLC: High performance liquid chromatography
IL-6: Interleukin-6
IL-8: Interleukin-8
iNOS: Inducible nitric oxide synthase
LDHA: Lactate dehydrogenase subunit A
LPS: Lipopolysaccharide
MIDs: Mass isotopologue distributions
MSTFA: N-methyl-N-(trimethylsilyl)-trifluoroacetamide
NF-κB: Nuclear factor-kappa B
NIST: National Institute of Standards and Technology
NSAIDs: Nonsteroidal anti-inflammatory drugs
OXPHOS: Oxidative phosphorylation
PBS: Phosphate buffer solution
PDK1: Pyruvate dehydrogenase kinase 1
PFKFB: Phosphofructo-2-kinase/fructose-2,6-bisphosphatase
PGE2: Prostaglandin E2
PI3K: Phosphoinositide 3-kinases
PMSF: Phenylmethylsulfonyl fluoride
pNPP: P-nitrophenyl phosphate
RA: Rheumatoid arthritis
RA-FLS: Rheumatoid Arthritis-Fibroblast-like synoviocyte
RI: Retention index
RPS9: 40S ribosomal protein S9
SDS-PAGE: Sodium dodecyl sulfate–polyacrylamide gel electrophoresis
TBS/T: Tris-buffered saline with Tween
TCA: Tricarboxylic acid
TMB: Tetramethylbenzidine
TMCS: Trimethylchlorosilane
TNF-α: Tumor necrosis factor-alpha
