# Supplementary figures and images for: Bovine tumor necrosis factor-alpha Increases IL-6, IL-8, and PGE2 in bovine fibroblast-like synoviocytes by metabolic reprogramming
Source: Sci Rep. 2023 Feb 24;13:3257. doi: 10.1038/s41598-023-29851-y (PMC9958177; doi:10.1038/s41598-023-29851-y)

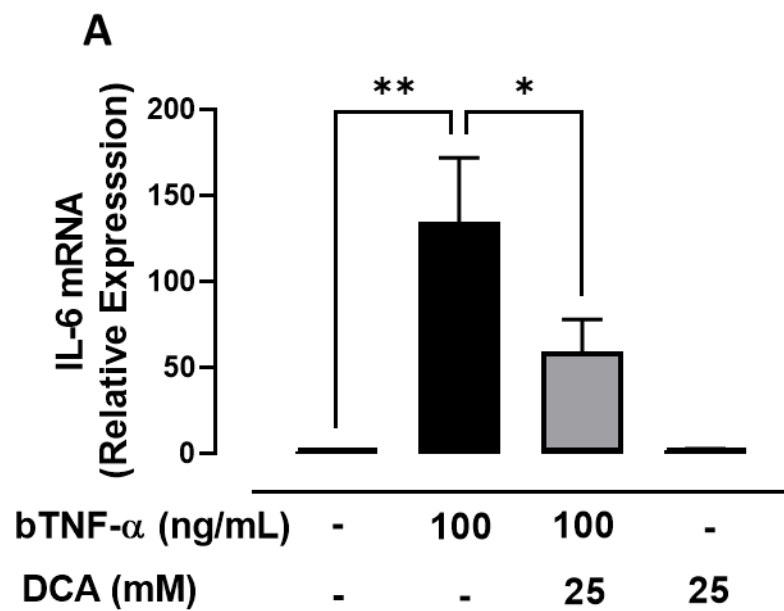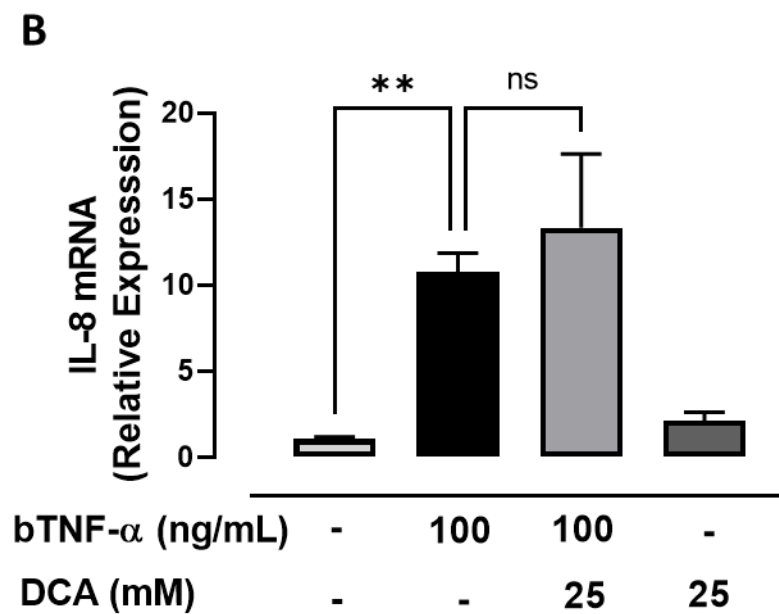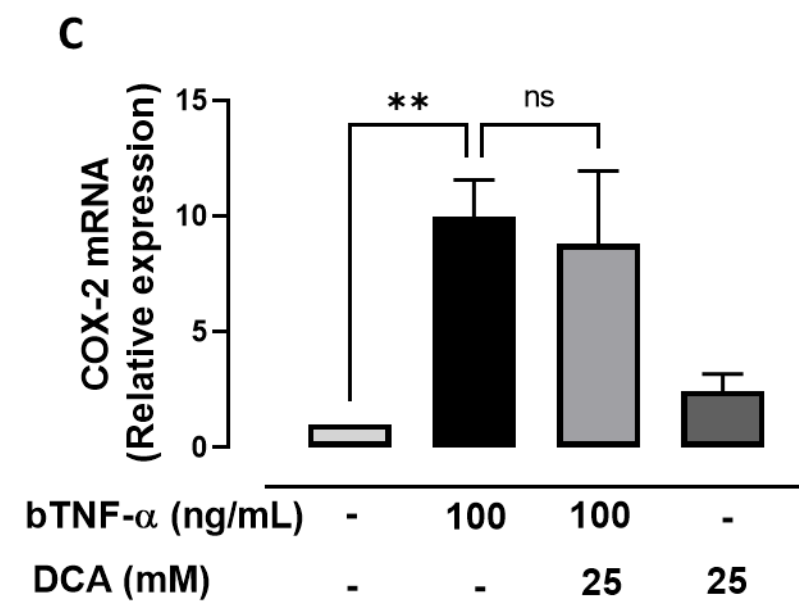

Supplement: Supplementary file 1 — Supplementary Information 1. [file 41598_2023_29851_MOESM1_ESM.pdf]

**A**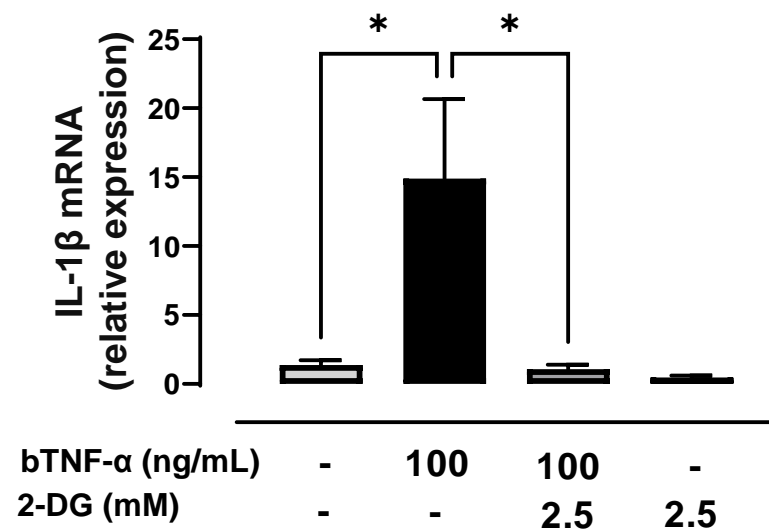**B**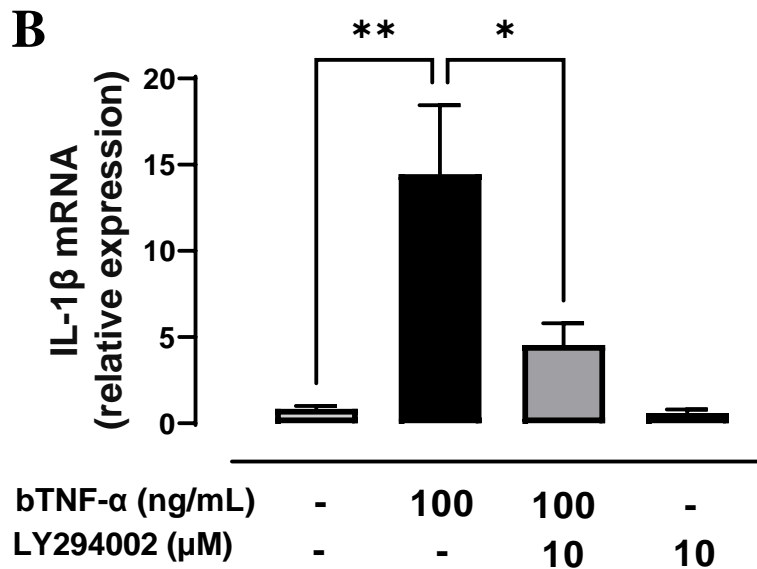**C**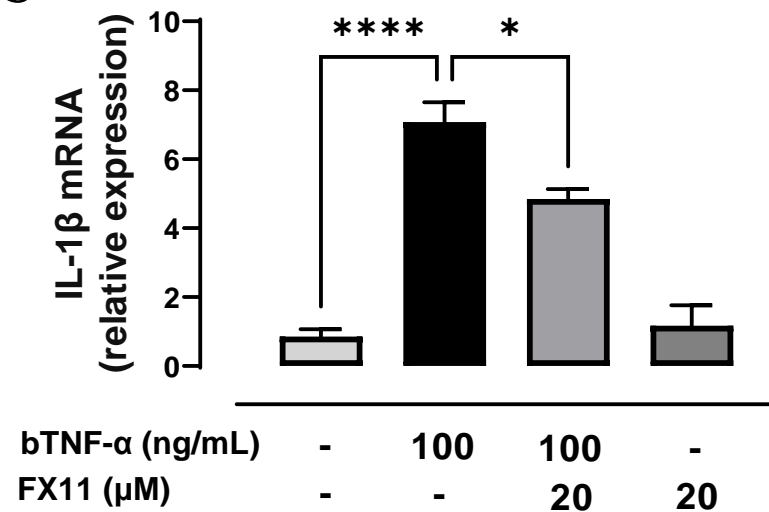**D**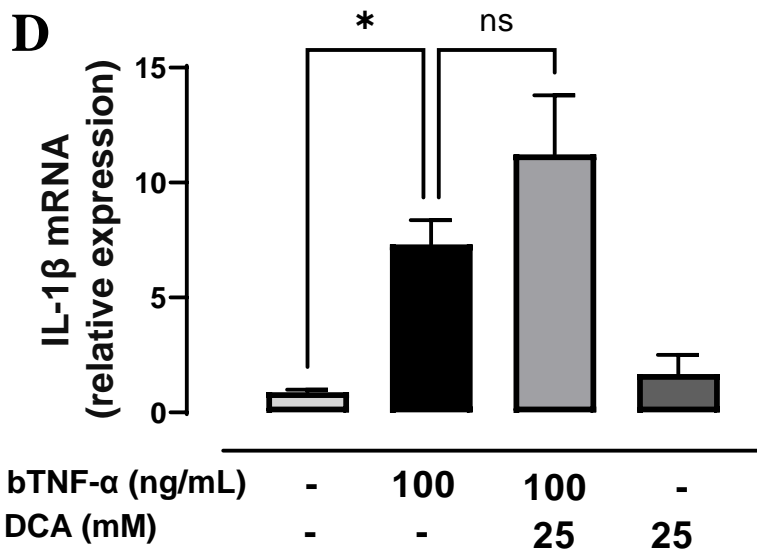**E**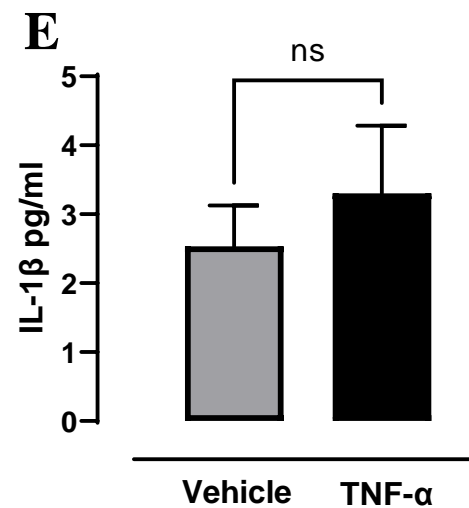

Supplement: Supplementary file 2 — Supplementary Information 2. [file 41598_2023_29851_MOESM2_ESM.pdf]

n1

n2

n3

n4

P-Akt

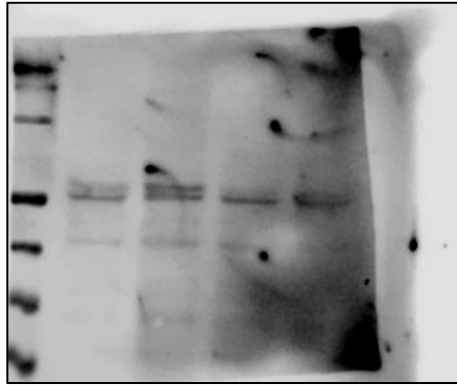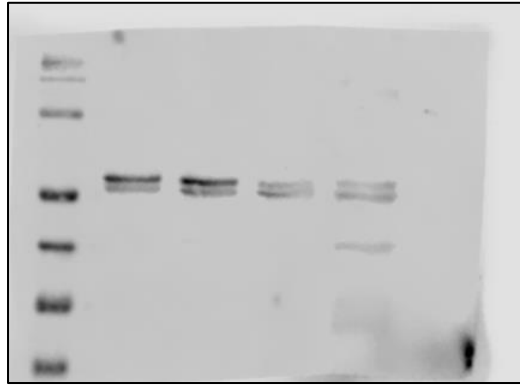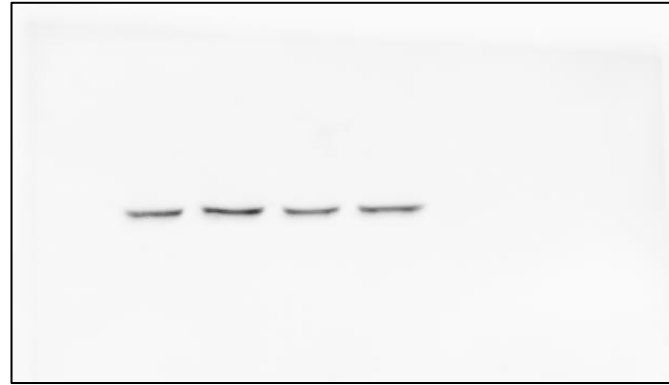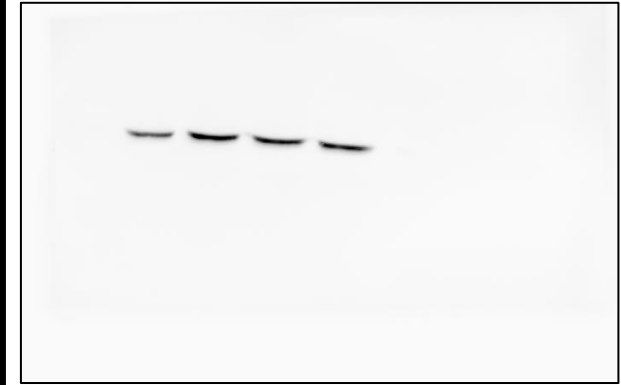

Akt total

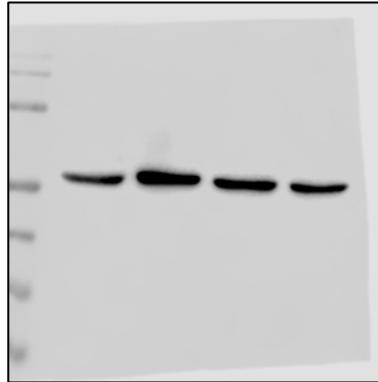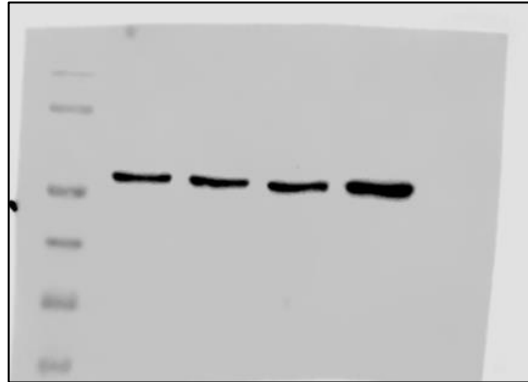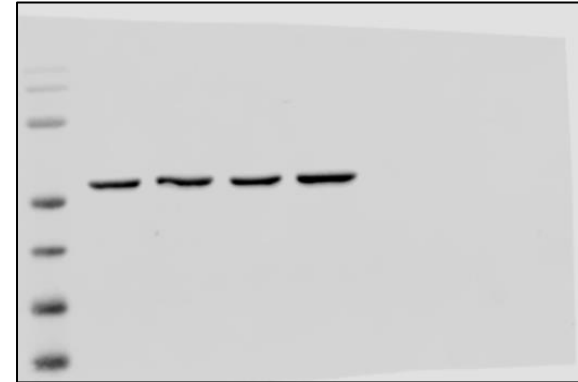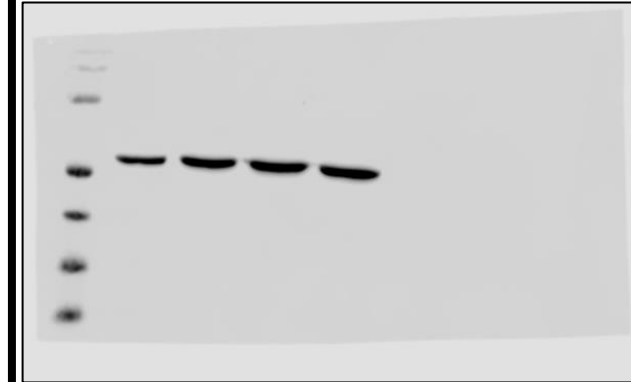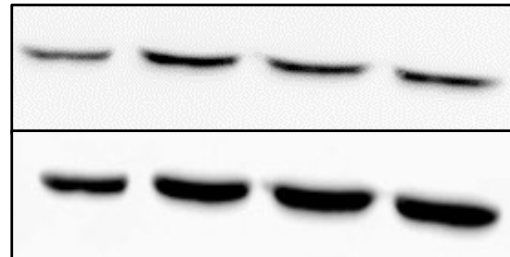

Supplement: Supplementary file 3 — Supplementary Information 3. [file 41598_2023_29851_MOESM3_ESM.pdf]
