# Supplementary material for: Bovine tumor necrosis factor-alpha Increases IL-6, IL-8, and PGE2 in bovine fibroblast-like synoviocytes by metabolic reprogramming
Source: Sci Rep. 2023 Feb 24;13:3257. doi: 10.1038/s41598-023-29851-y (PMC9958177; doi:10.1038/s41598-023-29851-y)
Supplement: Supplementary file 4 — Supplementary Information 4. [file 41598_2023_29851_MOESM4_ESM.docx]

**Supplementary Information**

Additional file 1 Fig. S1. Expression of IL-6 induced by bTNF-α is PDK dependent in bFLS. Relative expression of IL-6 (A), IL-8 (B), and COX-2 (C) in bFLS treated with DCA and stimulated with bTNF-α or vehicle. **p<0.01, and *p<0.05 with respect to the bTNF-α condition, n=4, each bar represents the mean ± SEM.

Additional file 2 Fig. S2. Expression and synthesis of IL-1β induced by bTNF-α in bFLS. Relative expression of IL-1β in bFLS treated with (A) 2-DG (B) LY294002 (C) FX11 and (D) DCA and stimulated with bTNF-α or vehicle. Synthesis of IL-1β induced by bTNF-α (E), *p<0.05, **p<0.01 and *****p*<0.0001 with respect to the bTNF-α condition, n=4, each bar represents the mean ± SEM.
